# Music programming for psilocybin-assisted therapy: Guided Imagery and Music-informed perspectives

**DOI:** 10.3389/fpsyg.2022.873455

**Published:** 2022-11-17

**Authors:** Catharina Messell, Lisa Summer, Lars Ole Bonde, Bolette Daniels Beck, Dea Siggaard Stenbæk

**Affiliations:** ^1^Neurobiology Research Unit, Copenhagen University Hospital Rigshospitalet, Copenhagen, Denmark; ^2^Anna Maria College, Institute for Music and Consciousness, Paxton, MA, United States; ^3^Center for Research in Music and Health, The Norwegian Academy of Music, Oslo, Norway; ^4^Institute for Communication and Psychology, Aalborg University, Copenhagen, Denmark; ^5^Department of Psychology, University of Copenhagen, Copenhagen, Denmark

**Keywords:** music program, psilocybin, guided imagery and music, music therapy, psychedelic-assisted psychotherapy

## Abstract

The psychedelic drug psilocybin has been successfully explored as a novel treatment for a range of psychiatric disorders. Administration of psilocybin requires careful attention to psychological support and the setting in which the drug is administered. The use of music to support the acute psychoactive effects of psilocybin is recommended in current guidelines, but descriptions of how to compile music programs for the 4–6 h long sessions are still scarce. This article describes the procedural steps and considerations behind the curation of a new music program, the Copenhagen Music Program, tailored to the intensity profile of a medium/high dose psilocybin. The method of Guided Imagery and Music is presented as a music therapeutic framework for choosing and sequencing music in music programming and the Taxonomi of Therapeutic Music is presented as a rating tool to evaluate the music-psychological intensity of music pieces. Practical examples of how to organize the process of music programming are provided along with a full description of the Copenhagen Music Program and its structure. The aim of the article is to inspire others in their endeavours to create music programs for psychedelic interventions, while proposing that an informed music choice may support the therapeutic dynamics during acute effects of psilocybin.

## Introduction

Music has been used in rituals across the world to achieve changes in consciousness throughout history, at times in combination with plants containing psychedelic compounds (Nettl, 2013). Literally translated from ancient Greek, psychedelic means “mind-revealing” (ψυχή = soul; δηλοῦν = to reveal) which is a name that hints to the therapeutic potential of these compounds if administered with careful deliberation. The classic psychedelics include primarily compounds that stimulate the brain’s serotonin system such as lysergic acid diethylamide (LSD), psilocybin, N,N-dimethyltryptamine and mescaline (Nichols, 2016). Of these, psilocybin, the prodrug of psilocin, is structurally similar to serotonin and produces its psychedelic effects through serotonin 2A receptor agonism (Kometer et al., 2012; Madsen et al., 2019). Psilocybin has been successfully explored as a novel therapeutic for a range of psychiatric disorders (Andersen et al., 2021) and is now tested in larger phase II studies of depression (e.g., ClinicalTrials.gov identifier: NCT03775200, C. G. I, 2022 ^2^). COMPASS Pathways is currently preparing a phase III trial testing psilocybin for treatment resistant depression. Recent clinical trials show promising results of treatment with psilocybin for patients with depression (Carhart-Harris et al., 2016, 2018a, 2021; Davis et al., 2021), addiction (Johnson et al., 2014; Bogenschutz et al., 2018, 2022), end-of-life anxiety (Grob et al., 2011; Griffiths et al., 2016; Ross et al., 2016) and obsessive–compulsive disorder (Moreno et al., 2006). Although psilocybin is the drug under investigation in these trials, it is widely assumed that the external environment (“setting”) and mind-set of participants (“set”) modulates the acute and long-lasting effects (Carhart-Harris et al., 2018b).

Music has been recommended as an integral part of psychedelic sessions since the early psychedelic studies (Gaston and Eagle, 1970). Today music is still recommended as part of the psychedelic setting (Johnson et al., 2008) and the role of music is becoming more salient in psychedelic research (Barrett et al., 2018). It is currently unknown, whether alternative approaches, e.g., silence or nature surroundings can be used interchangeably with music, but since most psychedelic studies use music as part of the intervention setting, gaining more insight into the facilitating potential of music is important. A recent meta-analysis including ten studies concluded that music which resonates with the patient’s experience supports self-exploration during the psychedelic experience (O’Callaghan et al., 2020). For example, liking the presented music is reported to promote safety and companionship (Belser et al., 2017; Noorani et al., 2018) and induce a sense of being on a personal journey (Gasser et al., 2014; Belser et al., 2017; Kaelen et al., 2018). Openness to and liking the presented music also correlate with the intensity of acute psychoactive effects of psilocybin and with better antidepressant treatment outcome (Kaelen et al., 2018), perhaps by enabling depressed patients to surrender and accept repressed emotions (Watts et al., 2017). These effects are likely compounded by the fact that psychedelic drugs themselves enhance the emotional and meaning-making response to music (Kaelen et al., 2015; Preller et al., 2017). Although music appears to be widely accepted as a central component in the psychedelic setting, the literature regarding appropriate choice of music for music programming in psychedelic therapy is surprisingly scarce. To gain a qualified perspective on the matter, researchers may look to the field of music therapy, which holds a wide body of knowledge regarding the therapeutic qualities of music and altered consciousness states.

As part of the early psychedelic research in the 1950’s and 60’s, music therapist Helen Bonny conducted research on the role of music in LSD sessions at Maryland Psychiatric Hospital (Bonny and Pahnke, 1972). In these sessions, she viewed the music as the primary mover of the therapeutic process, always present and actively influencing the patient (Summer, 1998). She and others found that music which matches the intensity of the drug effect could act as a non-verbal support with the capacity to facilitate relinquishment of control, emotional release, mystical experiences, and autobiographical insights (Eisner and Cohen, 1958; Gaston and Eagle, 1970; Bonny and Pahnke, 1972). Inspired by these early psychedelic studies, Bonny later developed the method of Guided Imagery and Music (GIM) (Bonny, 2002) after the prohibition of psychedelic drugs in 1970 (Oram, 2018). GIM is a receptive music psychotherapeutic method in which the patient listens to selected programs of classical music lasting 30–45 min, while exploring inner imagery with verbal guiding from the therapist (Bonny, 2002; Grocke, 2019). By use of relaxation techniques, the patient, who is laying down with eyes closed, is guided into a music-induced altered state of consciousness and invited to let the music *“take you where you need to go”* (Grocke, 2019, p.114). In GIM the music is understood to act as a co-therapist within the therapeutic triad of patient, therapist and music (Skaggs, 1992; Bonde, 2007, 43–74). Akin to the skilled therapist, music in GIM is understood to be a holding environment in which the music can regulate arousal and emotions (Grocke and Wigram, 2006; Koelsch, 2009), convey a sensed presence of an empathic ‘other’ (Summer, 1998; Levinge, 2015), facilitate embodiment (Beck, 2012; Bonde, 2017) and provide a sense of continuity and overall structure for experiences in altered states of consciousness (Lawes, 2017). We suggest that GIM may be a particularly relevant approach to consider when compiling a music program for use in psychedelic interventions. Based on the knowledge gained in GIM practice and from psychedelic research, we here describe our considerations and procedural steps for curating a novel music program for interventions with psilocybin.

With the intention of creating a novel music program for use in psilocybin sessions, i.e., the Copenhagen Music Program, which would accommodate a variety of cultural backgrounds, though primarily those of Northern Europe, we agreed on some overall criteria for the music program which were: (1) the music should reflect the intensity profile of a medium/high dose psilocybin, (2) the music should present cultural diversity of styles and genres, (3) vocal music pieces should avoid familiar languages, and (4) the music should avoid direct religious connotations. The procedural steps for creating the music program were inspired by (Bruscia, 2019) and will serve as the overall organisation of the article in four steps: (1) Setting up a structure for the music program, (2) Search and selection of music pieces, (3) Sequencing of music pieces for the music program, and (4) Indexing the intensity of music pieces in the music program. We hope that this format will provide a practical outline and inspire others in their endeavours to create music programs for psychedelic intervention.

### Setting up a structure for the music program

To meet the first criteria for the music program, we wanted it to reflect the experience of a medium/high dose of psilocybin, as this dose is commonly used in psychedelic research. Ingestion of a medium/high dose psilocybin elicits profound changes in consciousness, which lasts around 4–6 h and unfolds through a dynamic process in several phases (Leuner, 1962; Preller and Vollenweider, 2016). Recently, this process was modelled empirically in a study of healthy volunteers, which revealed three overall experiential phases: (1) the Ascent phase; (2) the Peak phase and (3) the Descent phase (Stenbæk et al., 2021). These Ascent, Peak and Descent contours were also observed in physiological responses to psilocybin such as blood pressure and hormonal secretion (Hasler et al., 2004; Holze et al., 2022). The phenomenology of the experience is described to change through these phases as a gradual build-up of effects, including perceptual, autobiographical and psychodynamic effects at lower intensities, over symbolic existential effects with transient ego-dissolution to deep integral levels of transcendent states at higher intensities. (Leuner, 1962; Preller and Vollenweider, 2016). We therefore created a working template of the music program, organized it in these three overall phases and applied the average time period of each phase, as measured by Stenbæk et al. (2021). Based on the phenomenology described above, we then created sub-phases with specific music-psychological opportunities for progress through the overall phase. Sub-phases of the Ascent phase were named: Opening, Onset, Build to peak and Going inside, sub-phases of the Peak phase were named: Confrontation & Surrender, Plateau and Transcendence, and sub-phases of the Descent phase were named: Emotional release, Reflection & Integration, Acceptance & Relief, Celebration and Landing & Return. Inspired by Hevners Mood Wheel (Hevner, 1936), which is often used in GIM to evaluate the emotional expression of a piece of music, adjectives referring to the music-psychological qualities of each sub-phase were noted; for example, music pieces for the sub-phase Confrontation & Surrender were noted with the adjectives: expansive, challenging, intense, mystical, sacred and cosmic, and music pieces for the sub-phase Acceptance & Relief were noted with the adjectives: lyrical, tender, holding, affectionate, and heartfelt. The creation of sub-phases and descriptions of the music-psychological qualities were inspired by the work of Bonny and Pahnke (1972) and Preller and Vollenweider (2016). However, due to the scarce available data regarding the temporal unfolding of experiential content during a medium/high dose of psilocybin, the authors have partly based the descriptions of sub-phases on their own clinical experience. These descriptions should therefore be seen as propositions, which need to be empirically validated in future studies. For an overview of our description of sub-phases and their corresponding music-psychological themes together with exemplary music pieces for each sub-phase see Table 1.

**Table 1:** Phases and sub-phases of the Copenhagen Music Program.

Phase	Sub-phase	Example music description	Psychological theme	Descriptive adjectives
Ascent Phase	1A Opening	No. 2: A Fairytale Slow pace, expressive melody, lyrical, repetition of theme in beginning and end, middle piece has a more unpredictable character	Inviting the listener to begin letting go of control	Calm, melodic, inviting
	1B Onset	No. 6: Optimist Opening with drum, increasingly more dynamic and rhythmical with more unpredictable structure and harmonic tensions.	Inviting the listener to deeper awareness and movement forward	Increasingly more rhythmical
	1C Build to peak	No. 9: O Magnum Mysterium Catholic church chant, choral piece, multiple harmonic layers with a wide span between low and high pitch register, slow pace with unfolding crescendos, no clear pulse, but instead carried by breath. Harmonic lines that move smoothly between major and minor.	Inviting the listener into a sacred and lofty space	Expansive, slow, sacred, solemn
	1D Going inside	No. 11: Gorecki 3rd Symphony, mvt 1 Orchestral music and soprano, spiralling harmonic progressions with more complex and unpredictable structure, dramatic, forceful, intense, long opening and ending of bass and cello	Inviting the listener into unknown domains with opportunity to face inner conflicting material	Dramatic, forceful, dark, pushy, insisting, ambiguous
Peak phase	2A Confrontation & surrender	No. 15: Sacred Words of Liberation Deep male voices and chants, bells, no pulse, electronic “weird” sounds. A sudden fall in pitch, floating tones in keyboard	Inviting the listener into expanded awareness of self, time and space	Expansive, intense, challenging, mystical, sacred, cosmic
	2B Plateau	No. 17: Bach Komm süsser Tod Orchestral music with repeated motives in melody, slow pace, steady pulse, tenderness in musical performance	Inviting the listener to be held softly	Lyrical, affectionate, soft, holding
	2C Transcendence	No. 21: Ohm Namah Shivaaya Electronic Eastern instrumentation and deep male voice, drones and overtones, low pitch tones, harmonies that slide between quarter tones. Moving from no pulse to pulse. Repetitive with “dragged” pulse. Changing rhythmical patterns and tempo towards the end	Inviting the listener to experience transcendence	Sacred, powerful, opening, mystical, spacious
Descent Phase	3A Emotional release	No. 26. Barber Adagio for strings String quartet, slow pace, soft beginning, Stepwise ascending motion in melody, building in intensity that culminates in string choirs high register climax followed by a full silence. Slow ending with prolonged tones and slowly fading accompaniment.	Inviting the listener to experience and release emotions	Empathic, thoughtful, melancholic, emotional, lofty
	3B Reflection & Integration	No 32. Tveitt O Be Ye Heartily Welcome Arrangement for piano. Soft opening and ending, ascending movement in accompaniment, musical suggestions and melodic dialog. Crescendo in the middle section with hard and forceful phrases.	Inviting the listener to dialog with inner psychological material	Hesitant, questioning, strange, thoughtful, contrasting
	3C Acceptance & Relief	No. 38 Manukyan: Where is she Armenian duduk. Lament over drone fifth. Eastern European folk music. Music with slow pace and lyrical melodic phrases	Inviting the listener to find self-care	Lyrical, affectionate, tender, heartfelt, holding
	3D Celebration	No. 42. Jobarthe Saya Rhythmical music, African style, female solo voice (alto) with band incl. Kora, flute, bass and female choir Repetitive, predictable structure, steady pulse, slow pace	Inviting the listener to embody and celebrate the endeavours accomplished	Celebrative, rythmical, vocal, repetitious, engaging and joyful,
	3E Landing & Return	No. 60. Pärt Spiegel im Spiegel Slow pace, melody in violin and a three-note accompaniment by piano. The melody ascends and is then mirrored by a descending melody line returning it back “home” to the central pitch. Simple and predictable harmonic structure. The performance is sensitive and present, like a meditative state.	Inviting the listener to land safely back into normal consciousness	Increasingly more calm, soft, steady and predictable

The table shows an overview of phases and sub-phases in the music program, their corresponding music-psychological themes and descriptions of music features. For each sub-phase an exemplary music piece is shown to illustrate how the music corresponds to the music-psychological themes.

### Search and selection of music pieces

Based on our working template, we began the process of searching for music by focusing on the few playlists for psychedelic research, that are currently available. One playlist was created for psilocybin therapy at Imperial College, London (Kaelen et al., 2018; Kaelin, n.d. ^3^) and consists of primarily neo-classical and ambient music with elements of jazz, classical and ethnic music. Two playlists were made for psilocybin therapy at Johns Hopkins University (Richards, 2003; Richards, 2015 ^4^; Strickland et al., 2020; Strickland, n.d. ^5^) of which one consists of primarily Western classical music and the other primarily of overtone music. A last playlist that was made for psilocybin research at the Chacruna Institute included more indie, new wave and post-rock (Thomas, n.d. ^6^). Apart from playlists for research, we listened to a variety of playlists made for psychedelic ceremonial work at retreat centres or within the psychedelic underground communities (i.e., Watts, n.d. ^7^; Rasa, n.d. ^8^), as well as a range of music programs made for GIM (Grocke, 2019; Bruscia and McShane (2014)). In our search for music, we primarily used music platforms like Spotify, ITunes and SoundCloud. Author 1 and 4 undertook the first selection of music pieces, of which each was categorized into one of the sub-phases. Each music piece that was deemed suitable according to the music-psychological qualities, underwent extensive critical listening for a range of specific details, such as the quality of sound in the specific recording and the musical performance, especially regarding presence, nerve, sensitivity, soulfulness and a general authenticity. In this respect qualities of singing voices and instruments were understood as important for the music to be engaging (Kaelen et al., 2018) and to embed qualities of an empathetic ‘other’ as described above (Summer, 1998; Levinge, 2015). We generally avoided well-known music- and vocal pieces with familiar language in order to offer a novel and curiosity-evoking, open experience with the music. Within the field of GIM, familiar languages are often avoided, as it may be experienced as too directive and thus distract the listener from experiencing the broader suggestions of the music’s inherent qualities (Bonny, 2002).

### Sequencing of music pieces for the music program

After critical listening to each music piece, we began the process of arranging the music pieces in meaningful sequences for the different sub-phases. Within the sequence of every sub-phase each piece of music would vary in regard to how its specific musical elements would serve the overall intent of the sub-phase; for example, the music could lead up to, prepare for, extend, give relief, add more variation or change direction from the overall music-psychological theme of a sub-phase (Bruscia, 2019, 401–12). The sequencing of music pieces involved a specification of the unique musical features and music-psychological quality of each selected piece in relation to the pieces surrounding it (Grocke, 1999; Bonde, 2007). To keep a record, the names of the music pieces and a description of their musical features and salient music-psychological qualities were noted in a table. To obtain a smooth transition between each of the music pieces, musical key, rhythm and sound in beginnings and endings were carefully examined and fitted together (Bruscia, 2019, 401–12), for example by connecting pieces in the same or related keys (according to the Circle of fifths) or by selecting pieces with the same basic note or one scale step up or down in modal music. Contrasts within and between music pieces and sections, such as instrumental/vocal, classical/electronic etc. were intentionally chosen to create a sense of opposing qualities, induce a sense of alertness or direct the listener in new directions (Bonny, 2002). In the same way that individual music pieces were carefully put together in sequences, sub-phases and overall phases were coordinated, until the program came together as a whole. To view the Copenhagen Music Program and the corresponding phases together with duration, tempo, key and genre of each music piece, see Table 2. The music program is available at: https://open.spotify.com/playlist/6QqL1JMtGAlw40kcMtBGDr?si=a47f1a017db74230 (Accessed January 15). After the first compilation of the music program by author 1 and author 4, all authors examined the music pieces and provided feedback both orally *via* online meetings and by commenting in the working template. During the process we repeatedly tested, revised and assessed the sequences to ensure that the original intention with the music program was met. This assessment also included approaching the music from a more affective-intuitive (Bonny, 1978) (as opposed to a cognitive, analytic) listening mode, by for example paying attention to subtle bodily and emotional reactions and by listening in an altered state of consciousness (Bonde, 2017, 269–277).

**Table 2:** The Copenhagen Music Program.

Phase	Sub-phase	*No.*	Music piece	Min.	Taxo-nomy	Key	Tempo (bpm)	Genre
Ascent Phase *Total time: 51:02 min.* .	1A Opening *Total time: 23:30 min.*	*1*	Pärt, A. (2019). Spiegel im Spiegel (violin and cello). [Recorded by S. Maer & S. Whitwell]. On *Classical chill: Cello*. ABC Classic. (Original work published 1978).	9:21	1	F Major	84	Classical
		*2*	Opsahl, J., & Opsahl, T. (2015). A fairytale. On *Unbroken dreams*. Heart to heart records.	4:31	2	G Major	92	Classical
		*3*	Økland, N., & Apeland, S. (2011). Sylkje-Per, variation. On *Lysøen (Hommage á Ole Bull).* ECM.	3:58	2	D Major	54	Traditio-nal
		*4*	Horn, P. (1989). Shah Jahan. On *Inside the Taj Mahal I & II*. Kuckuck.	5:40	3	A Major	55 (fluent)	New age
	1B Onset *Total time: 17:02 min.*	*5*	Einaudi, L. (2019). Gravity day 1. (Recorded by L. Einaudi, F. Mecozzi & R. Hasa). On *7 days walking Day 1–7*. Universal music group.	5:27	3	A Minor	60	Contem-porary
		*6*	Keating, Z. (2010). Optimist. On *Into the trees.* Vertebrae productions.	5:01	3	Eb Major	71/138 (shift)	Contem-porary
		*7*	Keating, Z. (2010). Escape artist On *Into the trees.* Vertebrae productions.	6:34	3	D Minor	78	Contem-porary
	1C Build to peak *Total time: 10:30 min.*	*8*	Gjeilo, O. (2016). Tundra. On *Ola Gjeilo*. Decca.	3:35	3	Bb Major	71	Classical
		*9*	Lauridsen, M. (1997). O magnum mysterium. (Recorded by Shaw chamber singers). On *A Robert Shaw christmas: Angels on high*. Telarc.	6:55	5	D Major	74	Classical
Peak phase *Total time: 79:52 min.*	2A Going inside *Total time: 30:33 min.*	*10*	Elgar, E. (2015). Enigma variations, Op. 36: Nimrod. (Recorded by Royal philharmonic orchestra). On *Last night of the proms*. Philips.	3:46	5	Eb Major	80	Classical	
		*11*	Gòrecki, H. (1992). Symphony no. 3, 1^st^ movement, Lento. (Recorded by London Sinfonia, D. Upshaw). Nonesuch records.	26:47	6	E Minor	52	Classical	
	2B Confrontation & surrender Total time: 30:19 min. .	*12*	Tchaikovsky, P. I. (1999). Hymn of the cherubim (From Liturgy of Saint John Chrysostom, opus 41). (Recorded by USSR Ministry of culture chamber choir). On *Universe 5*. Hearts of space.	7:36	9	Eb Minor	76	Classical	
		*13*	Poulenc, F. (2014). Stabat mater doloroso, I. (Recorded by Capella Amsterdam, Estonian philharmonic chamber choir, Estonian national symphony orchestra). On *Stabat mater*. Harmonia mundi.	4:50	9	A Minor	72	Classical	
		*14*	Pärt, A. (2010) Cantus *in memoriam* Benjamin Britten. (Recoded by Estonian National Symphonic orchestra). On *The very best of Arvo Pärt*. Emi.	6:48	9	A Minor	67	Classical	
		*15*	Lama Gyurme & Rykiel, J.-P. (2007). Sacred words of liberation. On *The lama’s chants - Roads of blessings/Songs of awe*. Last call records.	5:32	9	Eb Minor	62	New age	
		*16*	Hopkins, J. (2018). Feel first life. On *Singularity*. Domino recording.	5:33	9	C# Major	71	Contem-porary	
	2C Plateau *Total time: 9:29 min.*	*17*	Bach, J. S. (2016). Komm süsser Tod. (Arranged by L. Stokowski). (Recorded by Orchestre Métropolitain, Yannick Nézet-Séguin). On *Bonus track-Bach/Stokowski Choral 478*. Atma classique.	4:59	5	C Minor	70	Classical	
		*18*	Purcell, H. (2006). Dido and Aeneas. When I am laid in Earth. Dido’s lament, Z 626. (Arranged by L. Stokowski). (Recorded by Bournemouth symphony orchestra). On *Stokowski Bach transcriptions*. Naxos.	4:30	5	G Minor	73	Classical	
	2D Transcendence *Total time: 39:31 min.*	*19*	Von Bingen, H. (1997). O virtus sapiente (Arranged and recorded by Kronos quartet). On *Early Music (Lachrymæ Antiquæ)*. Nonesuch records.	4:32	5	E Minor	57	Classical
		*20*	Sawhney, N. (2015). Sacrifice. (Recorded by T. Tzarovska, J. Pook, V. Zivkovic, M. Pappenheim, & M. Yogeswaren). On *iTMOi (In the mind of Igor).* Pook music.	6:16	6	F Minor	72	Contem-porary
		*21*	Russill, P. (2007). Om namah Shivaaya. On *Shakti – Tantric embrace (Shakti yoga).* Relaxation company.	17:35	8	E Major	80/119	New age
		*22*	Haya Band & DaiQing, T. (2015). Ongmanibamai. On *Silent sky.* Wind music.	3:43	5	B Minor	73/101/ 121/71 (tempo shifts)	New age	
		*23*	Anilah (2014). Calling the others. On *Warrior*. Not on label. https://www.youtube.com/watch?v=JUP_7jo6vlA	6:26	6	F# Minor	120	New age
Descent Phase *Total time:* *208:37* *min.*	3A Emotional release *Total time: 45:35 min.*	*24*	Hoppe, M. & Wheater, T. (1999). The waiting. On *Afterglow*. Heart of space.	4:15	5	G Minor	61	Contem-porary
		*25*	Richter, M. (2014). Mercy. (Recorded by H. Hahn & C. Smythe). On *In 27 pieces: The Hilary Hahn encores.* Deutsche Grammophon.	5:32	4	Bb Minor	91	Contem-porary
		*26*	Barber, S. (2004). Adagio for strings, opus 11. (Recorded by New York Philharmonic Orchestra, Leonard Bernstein). *On Barbers adagio and other romantic favorites for strings.* Sony.	9:56	6	Bb Minor	74	Classical
		*27*	Elgar, E. (2001). Sospiri. (Recorded by English chamber orchestra, P. Goodwin). On *Elgar Nursery suite, Dream children, Serenade, and other works*. Harmonia mundi.	5:07	5	F Major	76	Classical	
		*28*	Pärt, A. (2006). Da pacem Domine. (Recorded by Estonia Philharmonic orchestra, P. Hillier). On *Arvo Pärt: Da pacem.* Harmonia Mundi.	5:43	5	D Major	84	Classical	
		*29*	Garbarek, J. (1994). Parce mihi Domine. (Arranged by J. Garbarek and Hillier ensemble). On *Officium.* ECM new series. (Composed by Christobal de Morales).	6:42	2	Bb Major	81	Contem-porary	
		*30*	Amar, A. (2006). Poem of the atoms II. On *Bab’ Azîz:The prince who contemplated his soul (soundtrack)*. Naïve.	2:20	5	B Minor	69	Contem-porary	
	3B Reflection & Integration *Total time: 11:27 min.*	*31*	Örvarsson, A., & Fang, S. (2020). *Engin Landamæri*. Flóra (Ost).	2:26	2	C Major	94	Contem-porary	
		*32*	Tveitt, G. (1997). Velkomne med æra - Welcome with honour. On *Piano music - 50 folk-tunes from Hardanger, Op. 150 / 24-part inventions, Op. 2 Nos. 1–12.* Naxos.	4:01	4	G Minor	80	Classical	
		*33*	Katchaturian, A. (1997). Gayane’s adagio. (Recorded by Skt. Petersborg state symphony orchestra). On *Ballet music from Gayane, Spartacus, Masquerade*. Naxos.	5:00	6	F Minor	66	Classical	
	3C Acceptance & Relief *Total time: 43:07 min.*	*34*	Arnalds, O. (2015). Reminiscence. On *The Chopin project*. Mercury classics.	4:28	5	C# Minor	56	Contem-porary	
		*35*	Mahler, G. (1992). Symphony no. 5, adagietto. (Recorded by Polish national radio symphony). On *Mahler*: *Symphony no. 5*. Naxos.	12:07	5	F Major	80	Classical	
		*36*	Elgar, E. (1997). Serenade for strings in E-minor, 2. Larghetto. (Recorded by Polish radio national symphony orchestra). On *The best of Elgar.* Naxos.	5:57	2	C Major	63	Classical	
		*37*	Amar, A. (2014). Pour une femme. On *Mediterranean. A sea for all (film track)*. Long distance.	4:49	5	C# Minor	92	Contem-porary	
		*38*	Manukyan, Y. (2000). Where is she. On *Armenian Duduk*. Karen studio/Believe SAS. https://www.melodlist.com/index.php?a=search&yti=i6TwTAA-7Qk	6:14	5	B Major	87	Traditio-nal	
		*39*	A Filetta. (2015) Sumiglia. On *Songs and polyphony from Corsica.* Digimusikka.	4:12	5	C# Minor	70	Traditio-nal	
		*40*	Kater, P., & Nakai, R. C. (2013). Offering. On *Ritual*. Mysterium music.	6:54	3	C Minor	80	Contem-porary	
	3D Celebration *Total time: 38:19 min.*	*41*	Cissoko, A., & Goetze, V. (2012). Amanké Dionti. On *Amanké Dionti*. Mótema.	6:31	2	D Minor	79	Traditio-nal	
		*42*	Jobarthe, S. (2020). Saya. On *Motherland - The score*. West African guild records.	3:53	2	C# Minor	91	Traditio-nal	
		*43*	Dreamers’ circus (2017). City gardens. On *Rooftop sessions*. GO´ Danish folk music.	5:06	2	G Major	81	Tradi-tional	
		*44*	Santaolalla, G. (2014). De Ushuaia a Quiaca. On *Ronroco*. Not on label.	2:54	2	G Minor	71	Traditio-nal	
		*45*	Curawaka (2018). He yama yo. On *Call of the wild*. Nixi music.	8:54	2	D Minor	67	New age	
		*46*	Scheurenbrand, R. (2010). Yemanja. On *Viento Bueno*. Rainer Scheurenbrand.	5:37	2	G Minor	54	New age	
		*47*	Arnal, M., & Barges, M. (2017). Ball del Vetlatori. On *45 cerebros 1 corazón*. Fina estampa.	5:24	2	C Minor	78	Traditio-nal	
	3E Landing & Return *Total time: 70:09 min.*	*48*	Delius, F. (2002). Aquarelle (Lento). (Recorded by Royal Northern Sinfonia). On *English string miniatures, vol. 4*. Naxos.	2:12	2	A Minor	72	Classical	
		*49*	Massenet, J. (1995). Sous les Tilleuls (from Scenes Alsaciennes). (Recorded by New Zealand symphony orchestra). On *Massenet orchestral suites.* Naxos.	4:59	2	Bb Major	63	Classical	
		*50*	Ashana (2009). Soulmerge. (Recorded with T. Barquee). On *Jewels of silence: Meditations on the chakras for voice and crystal singing bowls.* Angelic tones/ Barkawitz music.	9:36	1	E Major	63	New age		
		*51*	Portman, R. (2020). Much loved. On *Ask the river*. Node records.	4:22	2	E Major	64	New age		
		*52*	Satie, E. (2016). Gymnopedie no. 1, lent et douloureux. (Recorded by Olga Scheps). On *Satie*. Sony.	5:57	2	G Major	80	Classical		
		*53*	Saint-Saëns, C. (1998). The Swan (from Carnival of the Animals). (Recorded by Nadja Salerno-Sonnenberg). On *The most relaxing violon album in the world ever!* Virgin.	3:05	2	G Major	95	Classical		
		*54*	Winther, J. (2015). Om. On *Mantra*. Unisound.	5:45	1	D Minor	62	New age		
		*55*	Vasks, P. (2015). The fruit of silence. (Recorded by Latvian radio choir, S. Klava). On *The fruit of silence*. LMIC/SKANI.	7:27	2	Eb Major	73	Contem-porary		
		*56*	Bach, J. S. (2015). Adagio from toccata, adagio and fugue in C. (Recorded by Sinfonia Varsovia). On *Bach Konzerte und Transkriptionen.* Deutsche Grammophon.	3:26	2	A Minor	62	Classical		
		*57*	Gurdjieff, G. (2004). Prayer. (Recorded by A. Lechner & V. Tsabropoulus). On *Gurdijeff, Tsabropoulos*: *Chants, hymns and dances*. ECM new series.	3:50	2	F Minor	83	Classical		
		*58*	Schultz, M., & Sangha, M. S. (2014). Calma y tranquilidade. On *Simplementes satsang: Cantos et mantra (ao vivo)*. Simplementes Yoga.	7:26	2	D Minor	70	New age		
		*59*	Pepe, A. (2019). Felicia. On *Felicia*. IIP-DDS. https://music.youtube.com/playlist?list=OLAK5uy_kFWkTSGMAaWHwQY-tgf0XErNcshTTdGv4	2:15	1	G Major	61	Traditio-nal		
		*60*	Pärt, A. (1999). Spiegel im Spiegel (violin and piano). (Recorded by A. Malter, & V. Spivakov). On *Arvo Pärt: Alina*. ECM.	9:49	1	F Major	77	Classical

The table shows a full overview of music pieces in the music program and their corresponding taxonomy ratings together with duration, tempo, key and genre of each music piece. The music program is a mix of genres: 43.33% Classical, 23.33% Contemporary, 15% Traditional (world/folk music), and 18.33% New age music. The Ascent phase is characterized by pieces in Major keys with an increasing tempo (average 66 bpm), and a mix of genres. The Peak phase consists of pieces from mostly classical genres in Minor keys, with an average tempo of 72 bpm, including two new-age pieces that use tempo shifts up to 121 bpm. A mix of Major and Minor keys and genres and a descending tempo (average 61 bpm) are prominent in the longer Descent phase, where most Traditional and Film Music pieces are included.

### Indexing the intensity of music pieces in the music program

After the compilation of the music program, we applied a rating tool of music intensity developed in the field of GIM named the Taxonomy of Therapeutic Music (TTM) (Wärja and Bonde, 2014). The aim of applying this tool was to systematically explore whether the music program reflected the drug intensity profile of a medium/high dose of psilocybin (Stenbæk et al., 2021). The TTM consists of three prototypical music intensity profiles: (1) the Supportive, (2) the Mixed Supportive-Challenging, and (3) the Challenging. Each of these three main profiles is further divided into three sub-categories that express a continuum of intensity within the main profile. This makes it possible to rate a piece of music in one of nine sub-categories. The Supportive intensity profile consists of the following three sub-categories: (1) The supportive and safe field, (2) The supportive and opening field, and (3) The supportive and exploring field. The Mixed Supportive-Challenging intensity profile further consists of: (4) The explorative field with surprices and contrasts, (5) The explorative and deepening field, and (6) The explorative and challenging field, and the Challenging intensity profile consists of: (7) The rhapsodic field, (8) The field of metamorphosis and (9) The field of mystery and transformation (Wärja and Bonde, 2014). For a full description of music-psychological features of each of the three intensity profiles and their nine sub-categories with music examples, see Table 3.

**Table 3:** A taxonomy of therapeutic music – with examples from the GIM repertoire.

Intensity Profile	Sub-category	Description	Music examples	Taxonomy rating
Supportive	Supportive and Safe	Music that is reliable and predictable with no or few surprises. It will take you by the hand and lead you gently. Simplicity in musical elements and form, perhaps a solo instrument and/or one or two supporting instruments. Light moods only.	Stefan Nilsson: Nr 17, Wilmas Tema. Jan Johansson: Bandura.	1
	Supportive and opening	Music that can open up to one or two ‘tiny surprises’. Music with dialoguing instruments, possibly two different themes and at least two instruments.	Steve Dobrogosz: Mass and Chamber Music, Nr 13, Resting Place. Benny Anderssons Orkester, Nr 9, Sånger från andra våningen.	2
	Supportive and exploring	Music with some dynamic tension and complexity in texture and form. Gives further support for surrender and a possibility of exploring differences. Crescendos/decrescendos and accelerandos/ritardandos. Moderate harmonic tension.	Secret Garden: Papillon. Beethoven: Piano Concerto #5, Adagio.	3
Mixed Supportive-Challenging	The explorative field with surprises and contrasts	The music often presents a non-familiar soundscape, with surprising shifts in melody, harmony and specific instrumental texture. The musical course of events contains at least one major surprise, and there is moderate harmonic tension.	Bach: *Shepherd Song*. Respighi: *Gianicola*	4
	The explorative and deepening field	This is music that invites the listener into a welldefined emotional field, a certain mood or emotion, and holds the listener there, even though this can be challenging. The music is often in a minor or modal key, expressing a ‘dark’ atmosphere, typically through intense and expressive melody	Bach: *Mein Jesu*. Elgar: *Sospiri*. Mendelssohn: *5th symphony*, Andante.	5
	The explorative and challenging field	Music in this category offers some surprises and contrasts, often with arather high degree of melodic or harmonic tension. The balance is often obtained by letting the piece begin and end in a calm and supportive character/quality. The profile can also be movement towards a climax.	Bach/Stokowski: *Passacaglia and fugue in D*. Debussy: *Sirenes*. Brahms: *Violin Concerto*, 2nd movement. Rodrigo: *Concierto de Aranjuez*, 2nd movement.	6
Challenging	The rhapsodic field	The music is a sequence of often unrelated (or loosely related) musical ideas, presenting many different moods, textures, tempi and timbres. Ideas/elements can be quite elaborated or even improvisatory.	Bach: *Toccata and fugue in D*. Wagner: *Siegfried’s Funeral March*. Copland: *Appalachian Spring* (excerpt)	7
	The field of metamorphosis	Music is characterized by one or a few significant ideas that are elaborated in many different ways (shape, timbre, dynamics, tempo) and even transformed into something very different from the first form.	Ives: *The Unanswered Question*. Shostakovich: *5th symphony* (excerpt).	8
	The field of mystery and transformation	Music in this category cannot be generalised. However, it is often music that is intended to describe, express or facilitate transformative or mystic states of consciousness. The tempo is often slow, the mood predominantly dark, sombre or solemn.	Bach: *Crucifixus*. Rachmaninov: *Isle of the Dead*. Gorecki: *3rd symphony*, 2nd movement. Mahler: *Der Abschied* (excerpt from *Das Lied von der Erde*).	9

The table shows a full description of the three intensity profiles and their nine sub-categories in the taxonomy. Reprinted with permission from Bonde, L.O. and Wärja, M.

**Figure 1:**
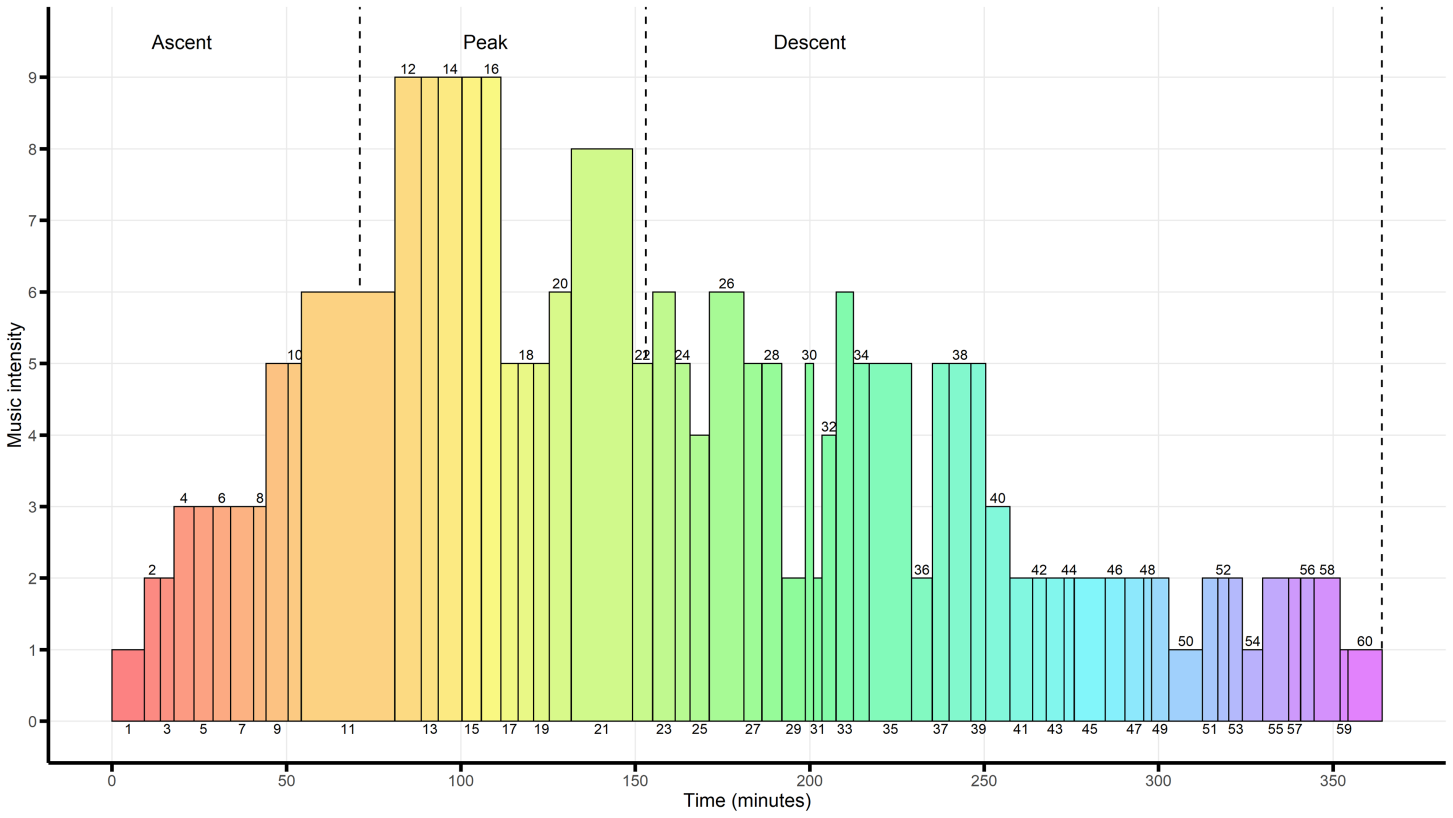
Intensity profile of the Copenhagen Music Program. Illustration of each of the 60 music pieces in the music program with intensity indicated on the y-axis and duration in time on the *x*-axis. Intensity of the music is measured by the Taxonomy of Therapeutic Music. Average time of the Ascent, Peak and Descent phases for a medium/high dose of psilocybin is indicated by the vertical dotted lines. Numbers in the figure refer to the place of each music piece in the music program. These numbers can also be found in Table 2 which provides a full overview of all the music pieces.

In TTM, music intensity is understood as a compound feature of the music, including its degree of complexity, dissonance, mood and quality (Wärja and Bonde, 2014). The nature of the taxonomy is phenomenological, with each sub-category representing typical patterns of musical form, texture, dynamics and melodic-harmonic development, all elements strongly influencing the listening experience (Jacobsen et al., 2019). TTM merges music analysis with salient psychological features of the music, and thus relates to the music psychology of Kurth (1931). Intensity rating of the music pieces according to the TTM was undertaken by author 3, who noted the musical elements and psychological qualities of each piece of music in a table. Author 4 then examined the intensity ratings and in case of any discrepancies between author 3 and author 4, they would together with author 1 discuss the music piece in question in order to reach concensus. An overview of TTM intensity ratings of each music piece can be found in Table 2. Figure 1 shows the intensity profile of the music program upon a background of the three overall phases of psilocybin drug intensity.

## Discussion

In this article we have described the curation of the Copenhagen Music Program by laying out a series of procedural steps and considerations rooted in music-psychological perspectives from the field of GIM. Overall, we found that these perspectives in GIM provide a language that unifies psychological concepts and musical analysis coupled with an understanding of how music can be experienced in altered states (Bonny, 2002; Grocke, 2019). We also became aware of important differences between selecting music for GIM and for a psychedelic music program. In GIM, music serves as the primary mover of the process, whereas music in a psychedelic session also supports and facilitates the effects of the drug. Therefore, unlike in GIM, the interaction between music and the drug has to be taken into consideration when compiling a psychedelic music program (Kaelen et al., 2015; Preller et al., 2017). This became evident, when we used the TTM to rate music pieces for the music program, where certain new age music pieces would be rated with medium intensity even though we had placed them in the end of the Peak phase (see Figure 1). For example, we would select music with “trance inducing” features such as repetitive rhythms, overtones or drones to support the listeners’ experience of the intensity of peak psilocybin effects, by providing them with a musical “anchor” (the drone and rhythm) and a sense of spaciousness (the overtones) (Hall, 2015) (e.g., music no. 20: Sawhney (2015) ^10^). In this sense the music was intended as a container of the drug effects and not as the primary mover of the process in that particular music piece. Importantly, TTM was created to assess the intensity of musical structures of classical music without intake of any drugs (Wärja and Bonde, 2014), which makes it suitable for GIM music programming. We suggest it as a valuable tool, which can be developed further for use in music programming for psychedelic intervention.

The method by which we rated the music pieces with the TTM can be criticised for not incorporating interrater reliability to substantiate the consistency of the ratings. As such it must be considered a tentative rating, that needs to be validated by other studies.

The temporal unfolding of phenomenological content with a medium/high dose of psilocybin is not well described (Stenbæk et al., 2021). Most of the available research focuses on retrospective summaries of the total psychedelic experience completed at the end of the session (e.g., Griffiths et al., 2011; Carbonaro et al., 2020). This impeded our ability to make empirically based decisions about music pieces at the more detailed level of sub-phases where we had to rely on more general phenomenological descriptions (Leuner, 1962; Preller and Vollenweider, 2016). More research is needed to inform these choices of music and we suggest that a neurophenomenological approach (Berkovich-Ohana et al., 2020) may be a good candidate for this type of temporal exploration of the psychedelic experience in future studies. We also see a need for randomised controlled studies evaluating the effect of music compared to no music on the unfolding psychedelic experience. Such knowledge would inform us about the role of music in a manner that controls for the effects of the drug.

Our approach to music programming for psychedelic interventions can be criticized for being too mechanistic and not taking the element of the therapeutic relationship and the patient’s choice of music into account (Read and Papaspyrou, 2021). In such a more music-centred approach (compared to a more patient-centred approach) the psychological-metaphorical structures of a piece of music are treated as having inherent causal potential for certain psychological processes (Schneck and Berger, 2005). However, we emphasize that the effect of music must always be considered in relation to the listener’s history, preferences and the cultural and social context of the listening experience (Summer, 2011; Bonde and Blom, 2016, 207–234). We do not understand the music effect as being causal in itself but view the role of the music as that of *inviting* the listener into a certain domain, which will be experienced in an individual manner by every listener (Bruscia, 2000). Importantly, a patient’s reactions to the music during a session can at times be an expression of a conflict that holds therapeutic significance [akin to the process of transference to a therapist (Bruscia, 1998)], in which case the therapist can support and encourage the patient to stay with the music and engage the conflict (Bonny, 2002; Beck, 2012; Grob and Grigsby, 2021).

When working with ethnic minorities or racial trauma, some authors have suggested that the music choice can amplify intercultural power dynamics in the therapeutic relationship (Michaels et al., 2018). Recent studies suggest that individualized music selections may hold some promise (Kaelen et al., 2018; Strickland et al., 2020), and within GIM, culturally adapted music programs have been shown to engage listeners in exploring and reconnecting to their cultural roots and identity (Swamy, 2018). However, the effect of using culturally adapted music programs in psychedelic-assisted therapy remains to be evaluated in future trials. If a therapist chooses to apply music from cultures foreign to them, it is advised that the therapist familiarize themselves with the function and cultural meaning of the music pieces, not to inflict unwanted associations in the listener (Short, 2005).

The Copenhagen Music Program was intended for possible use in psilocybin research, and although it was tailored to a medium-high dose of psilocybin, the procedures of curation can be modified and applied to music programming for other psychedelic substances, such as LSD, Ayahuasca or empathogens like MDMA.

## Conclusion

The procedural steps and music psychological considerations behind the creation of a new music program, i.e., the Copenhagen Music Program, for psilocybin treatment was described in the current paper. Music selection was based on perspectives from GIM, and the TTM was presented and discussed as a possible assessment tool of music intensity. We found that GIM-perspectives provided a helpful framework for understanding the possible therapeutic role of music in psychedelic interventions. This approach may inspire others in curating music programs for psychedelic therapy and research. More music programs and theory developments are needed along with empirical testing of music programs to gain a better understanding of how music may complement and support psychedelic intervention.

## Data availability

The original contributions presented in the study are included in the article/supplementary material, further inquiries can be directed to the corresponding author.

## Author contributions

CM, DS, and BB contributed to the conception of the work and wrote the first draft of the manuscript. CM and BB conducted the first working template of the music program. CM, LS, LB, BB, and DS contributed to the analysis and interpretation of the individual music pieces and the final compilation of the music program, critically reviewed the manuscript and approved the final submitted version. All authors contributed to the article and approved the submitted version.

## Funding

The project was supported by The Independent Research Council Denmark (grant number 9058-00017A) and The Health Foundation (grant number 21-B-0358).

## Conflict of interest

The authors declare that the research was conducted in the absence of any commercial or financial relationships that could be construed as a potential conflict of interest.

## Publisher’s note

All claims expressed in this article are solely those of the authors and do not necessarily represent those of their affiliated organizations, or those of the publisher, the editors and the reviewers. Any product that may be evaluated in this article, or claim that may be made by its manufacturer, is not guaranteed or endorsed by the publisher.

## Footnotes

^1^https://clinicaltrials.gov/ct2/show/NCT03775200?term=psilocybin+phase2&type=Intr&cond=Depression&age=1&phase=1&draw=5&rank=13.

^2^ https://open.spotify.com/playlist/6q0rnAIIxokcRlu6vRJPNX?si=6ed1a84816144904

^3^ https://open.spotify.com/playlist/5KWf8H2pM0tlVd7niMtqeU?si=188ef52ff5db4b77

^4^ https://open.spotify.com/playlist/40OOW1CBqejT3j0r56FDTS?si=481b6d13ecf04c0b

^5^ https://open.spotify.com/playlist/7yNhMh9EPndPBjle9Gx4Gx?si=b3b316cd0c7c413b

^6^ https://open.spotify.com/playlist/1LBcs5ACHGjtmRs4vAnmLh?si=ad4b0ff929384a1d

^7^ https://soundcloud.com/search?q=This%20Journey%20We%20Take

^8^ https://open.spotify.com/track/7iO9HcAP57k4XzJdA4k5Ws?si=eda08294469b43f4
